# Clinical features and gene mutational spectrum of *CDKL5*-related diseases in a cohort of Chinese patients

**DOI:** 10.1186/1471-2350-15-24

**Published:** 2014-02-25

**Authors:** Ying Zhao, Xiaoying Zhang, Xinhua Bao, Qingping Zhang, Jingjing Zhang, Guangna Cao, Jie Zhang, Jiarui Li, Liping Wei, Hong Pan, Xiru Wu

**Affiliations:** 1 Department of Pediatrics, Peking University First Hospital, Beijing 100034, China; 2 National Institute of Biological Sciences, Peking University, Beijing 100871, China

**Keywords:** *CDKL5* mutations, Early-onset epileptic encephalopathy, X chromosome inactivation

## Abstract

**Background:**

Mutations in the cyclin-dependent kinase-like 5 (*CDKL5*) (NM_003159.2) gene have been associated with early-onset epileptic encephalopathies or Hanefeld variants of RTT(Rett syndrome). In order to clarify the *CDKL5* genotype-phenotype correlations in Chinese patients, *CDKL5* mutational screening in cases with early-onset epileptic encephalopathies and RTT without *MECP2* mutation were performed.

**Methods:**

The detailed clinical information including clinical manifestation, electroencephalogram (EEG), magnetic resonance imaging (MRI), blood, urine amino acid and organic acid screening of 102 Chinese patients with early-onset epileptic encephalopathies and RTT were collected. *CDKL5* gene mutations were analyzed by PCR, direct sequencing and multiplex ligation-dependent probe amplification (MLPA). The patterns of X-chromosome inactivation (XCI) were studied in the female patients with *CDKL5* gene mutation.

**Results:**

De novo *CDKL5* gene mutations were found in ten patients including one missense mutation (c.533G > A, p.R178Q) which had been reported, two splicing mutations (ISV6 + 1A > G, ISV13 + 1A > G), three micro-deletions (c.1111delC, c.2360delA, c.234delA), two insertions (c.1791 ins G, c.891_892 ins TT in a pair of twins) and one nonsense mutation (c.1375C > T, p.Q459X). Out of ten patients, 7 of 9 females with Hanefeld variants of RTT and the remaining 2 females with early onset epileptic encephalopathy, were detected while only one male with infantile spasms was detected. The common features of all female patients with *CDKL5* gene mutations included refractory seizures starting before 4 months of age, severe psychomotor retardation, Rett-like features such as hand stereotypies, deceleration of head growth after birth and poor prognosis. In contrast, the only one male patient with *CDKL5* mutation showed no obvious Rett-like features as females in our cohort. The X-chromosome inactivation patterns of all the female patients were random.

**Conclusions:**

Mutations in *CDKL5* gene are responsible for 7 with Hanefeld variants of RTT and 2 with early-onset epileptic encephalopathy in 71 girls as well as for 1 infantile spasms in 31 males. There are some differences in the phenotypes among genders with *CDKL5* gene mutations and *CDKL5* gene mutation analysis should be considered in both genders.

## Background

Mutations in the cyclin-dependent kinase-like 5 gene (*CDKL5*, OMIM 300203) have been described in *CDKL5*-related disorders including early-onset seizure variant of rett syndrome and early-onset epileptic encephalopathies [1]. Although *CDKL5* mutation screenings are generally performed in cohorts of Rett syndrome (RTT) or a variant of it, individuals with *CDKL5* mutation present their unique characteristics. Intractable epilepsy that resistant to multiple antiepileptic drugs (AEDs) often starts within 6 months of age and is associated with severe mental retardation without obvious period of regression. Clinical manifestations of subsequent cases with *CDKL5* mutation closely resemble some features of RTT including hand stereotypies, autistic-like features, scarce acqusition of language and concious hand use. Accumulated evidence show that phenotype of *CDKL5*-related disorders overlaps with early-onset seizure variant of RTT (RTT, OMIM 312750) and early-onset epileptic encephalopathies with X-linked infantile spasms (ISSX,OMIM308350) [2-7]. Nowadays, the phenotypic spectrum range of *CDKL5*-related diseases has expanded. More than 80 mutations in *CDKL5* had been reported, in which 10% females were found with early-onset seizure variant of RTT, early infantile epileptic encephalopathy-2 and myoclonic encephalopathy [8-10]. Different *CDKL5* mutation types include missense mutations, nonsense mutations, splicing mutations and deletion/insertions causing frameshifts and premature truncation [11]. Large rearrangement of the *CDKL5* gene has been detected by multiple ligation-dependent probe amplification (MLPA) [7,10,12]. The reported mutations locate almostly across all exons of *CDKL5* gene and disrupt either the catalytic domain or the C-terminus domain of CDKL5 protein. Recent data suggests that missense mutations located in the catalytic domain of *CDKL5* could exhibit more severe clinical manifestation than that caused by truncated mutations located in the C-Terminal of *CDKL5*[13]. However, further investigation is still needed to elucidate the relationship between clinical severity and mutation types and locations. Although most of the *CDKL5* mutations are found in females, they also present in some males. Recognition of male *CDKL5* mutation carriers broadens the spectrum of *CDKL5*-related diseases and suggests that *CDKL5* mutation screening should be carried out in both genders [14].

The purpose of this study is to delineate the clinical manifestation of Chinese patients with *CDKL5*-related diseases, identify the *CDKL5* genotype-phenotype correlation in both genders and broaden the spectrum of *CDKL5* mutations. Although information of Japanese Asian and Indian Asian have been published [14,15], the similar data are scarce in Chinese Asian. This work is the first study of *CDKL5*-related diseases in a large cohort of Chinese patients.

## Methods

### Patients

One hundred and two Chinese patients, including 71 females and 31 males, were recruited after informed consent obtained from the parents of the patients. The median age was 1 year and 4 months (ranged from 1 month to 12 years). Among them, 64 cases were diagnosed with early-onset epileptic encephalopathies, including 36 with infantile spasm, 8 with Ohtahara syndrome, and 20 with unknown epileptic syndrome. The seizures of the patients presented within six months of life without known causes in all cases recruited in our cohort. Thirty-eight cases were diagnosed with RTT, including 16 with classical RTT, 10 with congenital RTT, 3 with preserved speech variant RTT and 9 with Hanefeld variant of RTT. *MECP2* mutations were negative in all the RTT patients. The clinical information was collected from family questionnaire as well as clinicians’ reviews. Laboratory investigations included blood and urine amino acid and organic acid screening, brain MRI and electroencephalogram (EEG). Ethical approval was obtained from Clinical Research Ethics Committee, Peking University First Hospital.

#### *CDKL5* gene mutational analysis

Genomic DNA was extracted from the peripheral blood leukocytes of the patients and their parents. *CDKL5* gene (NM_003159.2) mutations were analyzed by polymerase chain reaction (PCR) and direct sequencing. PCR was carried out in 25 ul reaction system with 2 × GC Buffer I 12.5 ul, deionized H_2_O 4.5 ul, 5′ primer 2 ul (5Pmol), 3′ primer 2 ul (5Pmol), dNTPs 2ul, platinum rTaq DNA polymerase (Invitrogen) 1ul and DNA template 1 ul. The annealing temperature was 59°C for exon1-21 except for exon 5 with 57°C. Primer sequences and polymerase chain reaction conditions are available upon request. GenBank accession number NM_003159.2 was used as the DNA reference sequence. The nomenclature used follows the HGVS mutation nomenclature guidelines (http://www.hgvs.org). Pathogenicity prediction of previously unreported variants was performed using Alamut version2.0 (Interactive Biosoftware, Rouen, France). When no mutation was founded, MLPA (SALSA MLPA kit P189 *CDKL5*, MRC-Holland, Amsterdam, Holland) was performed to detect large deletions or duplications of *CDKL5* gene, according to the previous reports [16,17].

#### The pattern of XCI in female patients with *CDKL5* mutation

The pattern of XCI was analyzed in the female patients with *CDKL5* gene mutations, according to the procedures described by Allen et al. [18]. XCI was considered skewed if the ratio was ≥80:20, and considered extremely skewed if the ratio was ≥85:15.

## Results

### *CDKL5* gene mutations

De novo *CDKL5* gene mutations were found in ten patients including one missense mutation (c.533G > A, p.R178Q) which had been reported, two splicing mutations (ISV6 + 1A > G, ISV13 + 1A > G), three micro-deletion (c.1111delC, c.2360delA, c.234delA), two insertions (c.1791 ins G, c.891_892 ins TT in a pair of twins) and one nonsense mutation (c.1375C > T, p.Q459X). Among the ten patients, nine were females (12.6%, 9/71) and one was male (3.22%, 1/31). The mutation rate was 9.8% (10/102). Out of the patients who carried a mutation, one male (2.77%, 1/36) case was diagnosed with infantile spasm, followed by seven female (77.7%, 7/9) with the Hanefeld variant of RTT, two female (10%, 2/20) with unknown epileptic syndrome and none (0%, 0/8) with Ohtahara syndrome. No mutation was found in their parents (Table 1).

**Table 1:** Detailed clinical manifestations, mutations and ration of XCI of patients

**NO.**	**Patient 1**	**Patient 2**	**Patient 3**	**Patient 4**	**Patient 5**	**Patient 6 and 7**	**Patient 8**	**Patient 9**	**Patient 10**
Age	2 yrs	3 yrs	3 yrs	4 yrs	5 yrs	2 yrs and 6 mo	5 mo	13 mo	2 yrs and 9 mo
Gender	F	F	F	F	F	F	M	F	F
Seizure onset	22 ds	10 ds	1.2 mo	3.3 mo	1 mo	2.3 mo	2 mo	1.2 mo	1.3 mo
Partial seizures	22 ds- now	10 ds-2 yrs	1.2 mo- now	3.3 mo-3 yrs	1 mo-now	2.3 mo-now	2 mo-now	1.2 mo-now	1.3 mo-now
Spasm	2.2 mo-now	2 mo-now	1.5 mo-now	3.7 mo-3 yrs	1.5 yrs-now	3 mo-now	2 mo-now	-	2.5 yrs-now
Myoclonic seizures	-	2 yrs-now	-	-	-	-	-	-	2.8 yrs-now
Tonic seizures	-	2 yrs-now	-	-	-	-	-	-	2.8 yrs-now
Drug resistance	+	+	+	+	+	+	+	+	+
Hypsarrhythmia	**-**	**+**	**-**	**-**	+	+	+	-	-
Deceleration of head growth	+	+	+	+	+	+	+	+	+
Hypotonia	+	+	+	+	+	+	+	+	+
Limited hand skills	+	+	+	+	+	+	+	+	+
Stereotypies	+	+	+	+	+	+	-	+	+
Sit unaided	16 mo	11 mo	18 mo	24 mo	6 mo	6 mo	-	-	-
Walk (ever)	-	-	-	-	A few steps	_	-	-	-
Speech	Absent	One word	Absent	Absent	Absent	Absent	Absent	Absent	Absent
Autistic features^**a**^	+	+	+	+	+	+	+	+	+
Autonomic features^**b**^	NA	+	NA	+	+	+	-	+	+
MRI or CT	-	-	-	-	-	-	-	-	-
*CDKL5* mutations	c.1111 delC	ISV13 + A > G	c.1791 insG	ISV6 + 1A > G	c.1375C > T;	c.891_892ins TT	c.533G > A;	c.2360delA	c.234delA
Position of mutatins	C-terminal	C-terminal	C-terminal	Catalytic domain	C-terminal	Catalytic domain	Catalytic domain	C-terminal	Catalytic domain
XCI ratio	50:50	63:37	55:45	53:47	58:42	60:40/56:44	-	52:48	54:46

*Abbreviations:* EOEE, early onset epileptic encephalopathy; Hanefeld RTT, Hanefeld variant of Rett syndrome; IS, infantile spasms; F, female; M, male; yrs, years; mo, months; ws, weeks; ds, days; HC, head circumference; cm, centimeter; NA, nonavailable.

^**a**^autistic features include avoidance of eye gazing, reduced social interaction.

^**b**^autonomic features include breathing dysrhythmia, hypalgesic, bruxism, cold extremities.

### XCI pattern

All the nine female patients with *CDKL5* gene mutations had random X chromosome inactivation. The ratios were 50:50, 63:37, 55:45, 53:47, 58:42, 60:40, 56:44, 52:48 and 54:46, respectively (shown in Table 1).

### Clinical manifestation

Among the ten patients with *CDKL5* gene mutation in the cohort, phenotype consisted of 7 females with Hanefeld variant of RTT, 1 male with infantile spasms and 2 females with early onset epileptic encephalopathy at the first referral. All the ten patients were attacked by early onset seizures before 4 months of age (from 10 days to 100 days after birth). Various seizure types presented in the course of the disease including epileptic spasms, partial seizure, myoclonic seizure and tonic seizure. However, all the patients were initially attacked by partial seizure, and then it transformed to spasm or to the case that including both partial seizure and spasm after a period of time (10 days to 2.4 years). In the follow-up evaluation, myoclonic seizure and tonic seizure presented together with the original seizure types in two patients. All types of seizure in the patients with *CDKL5* gene mutation were intractable and resistant to the antiepileptic drugs (AEDs). Hypsarrhythmia was identified on EEG records in five patients. Some Rett-like features presented in 9 female patients, such as the presence of hand-mouthing, hand washing or clapping became apparent in the first year of life in all of the female patients. All the patients with *CDKL5* mutation showed severe psychmotor developmental delay. In addition, hypotonia, poor to absent acquisition of language, limited hand skills, poor eye contact, autonomic dysfunction features and autistic symptoms were the common features in all the patients. MRI, blood and urine amino acid and organic acid were normal in all the patients. Detailed information may be found online in the supporting information part (Additional file 1).

## Discussion

Kalscheuer reported two unrelated girls with infantile spasms and mental retardation whose *CDKL5* gene were disrupted by different balanced X-autosome translocations in 2003 [19]. Since then, only about 80 cases with *CDKL5* mutations have been reported around the world. The patients have various phenotypes including early-onset seizure variant of RTT, X-linked infantile spasms, early infantile epileptic encephalopathy-2, autism spectrum disorders (ASDs) and Rett-like syndrome or Angleman-like syndrome [20-24].

In this paper, we submit a report on ten more cases of *CDKL5*-related diseases, which is the first report on Chinese asian. Nine females and one male with *CDKL5* gene mutations are found in 102 patients with early-onset epileptic encephalopathy and RTT. The overall mutation rate of *CDKL5* is 9.8% (10/102), which is similar to that of previous studies [6,9,14]. Among the patients with *CDKL5* gene mutations, 2 are diagnosed with early onset epileptic encephalopathy, 1 with infantile spasms and 7 with early-onset seizure of RTT syndrome when they were first referred to the clinician. It is worth noting that the two females with early onset epileptic encephalopathy present many characteristics of early-onset seizure of RTT syndrome in the follow-up assessment. The *CDKL5* gene mutations in our patients include missense mutation (1/10), nonsense mutation (1/10), splicing mutations (2/10) and insertion/deletion mutation (6/10) causing frameshifts. No large rearrangement of the *CDKL5* gene is found. All the mutations are proved to be de novo, and no mutations are further detected in their parents. Among the detected mutations in this paper, p.R178Q carried by a boy and a splicing mutation IVS6 + 1G > A carried by a girl are reported previously [3,4,16,21].

Epilepsy is the core symptom of the patients with *CDKL5* mutation. Bahi-Buisson N [13] concluded three successive stages of the epilepsy: early epilepsy (onset 1-10 weeks) with normal interictal EEG (stage I); epileptic encephalopathy with infantile spasms and hypsarrhythmia (stage II); refractory tonic seizures and myoclonia (stage III). In all our patients, the clinical manifestations are seizures, occurring in a range from 10 days to 100 days, the average of which is 47 days. The initial seizure type of all the patients is partial seizure. Epileptic spasm presents in 9 patients several days to 2 years after the onset of partial seizure. Other seizure types including myoclonic seizure, tonic seizure and atypical absence presents in two patients. Hypsarrhythmia or atypical hypsarrhythmia are found in five patients. The epileptic events are resistant to all treatments including the antiepileptic drugs, ACTH and ketogenic diet. In all our patients, frequent seizure continues even after the extensive treatment except for case 4 who was seizure-free 3-years later.

All the patients show severe psychmotor developmental delay. Seven patients could sit unaided, the time delayed in 6 patients is in the range from 8 months to 24 months, the average which is 12.5 months. The other three cases aged from 5 months to 2 years and 9 months could not sit at all. Nine patients could not walk. All the female patients with *CDKL5* mutation show some Rett-like features such as stereotypic hand movements. The presence of hand-mouthing, hand washing or clapping becomes apparent in the first year of life in seven patients. In addition, hypotonia, limited hand skills, poor eye contact and autistic symptoms are the common features of all the patients. Although the symptoms of our patients with *CDKL5* mutations overlap with some features of Hanefeld variant of RTT, recent reports have clarified that *CDKL5*-related disorder should be considered as separate from RTT, rather than another variant [25,26].

Despite the common features shared by patients with *CDKL5* mutation of both genders, there are some phenotypic differences between females and males [3,16]. Seven males with *CDKL5* mutations have been reported so far, which are much less than females. Bahi-Buisson N conducted a comparison of clinical features between both genders. The results showed that none of the reported males acquired language skills (0% vs 12.3%) or ambulation with or without aid (0% vs 32.4% ) compared to female patients. Of greater interest is that Rett-like features such as hand stereotypies have scarcely been reported in males with *CDKL5* mutations [3,16,27-29]. In agreement with the reported male characteristics, the male patient aged 5 months in our cohort manifests early-onset seizures, hypotonia, poor eye contact and poor response to antiepileptic drugs. He does not acquire hand skills and never develop apparent hand stereotypies as female patients. The clinical manifestations still need further observations due to the young age of the boy. The most recent reports indicates that there are no differences in clinical severity between both genders with *CDKL5* mutation [8], and more male cases are necessary to support this conclusion.

*CDKL5* encodes a serine threonine kinase protein composed of a catalytic domain and a long C-terminal extension. Catalytic domain regulates the catalytic activity of *CDKL5* gene and the C-terminal involves in sub-nuclear localization of the protein and negatively regulates the catalytic activity [30]. The clinical severity may be associated with the location and the type of mutations, but the genotype-phenotype correlation still remains obscure [22]. It has been reported that some recurrent mutations in N-terminal catalytic domain such as p.Arg59X, p.Arg134X, p.Arg178Trp/Pro/Gln, c.145 + 2 T > C and frameshift mutations in the C-terminal region such as c.2635_2636del CT cause more severe phenotype, which is manifested by earlier onset, infantile spasms and hypsarrhythmia, refractory epileptic encephalopathy, microcephaly, and inability to walk [31]. The patients with p.Ala40Val mutation which affects ATP-binding site in catalytic domain, showed mild phenotype (walked unaided, normocephaly, better hand use ability, and less frequent refractory epilepsy), compared to those with other *CDKL5* mutations [5,6,13]. Russo et al. proposed that patients with stop codon mutations have a milder phenotype than those with missense or splicing mutations [12]. In our cohort, there are no obvious differences between catalytic domain (patient 4, 6 and 7, 8 and 10) and C-terminal (patient 1, 2, 3, 5 and 9) domain. Patient 4 with splicing mutation shows seizure-free after 3-year old. She is the only patient who could walk a few steps and sit unaided at 6 months. Therefore, in our study we cannot develop any clear relationship between the clinical manifestation and the type of *CDKL5* mutations, due to the limited number of mutation samples in our study. The pattern of XCI is known as the important modulator of the phenotype in X-link genetic diseases. Kalscheuer VM et al. [19] found that the normal X chromosome in two females with heterozygous *CDKL5* mutations was functionally absent, suggesting *CDKL5* located in X gene was subject to inactivation in female somatic cells. Tao et al. [20] showed that variable expression of the wild-type CDKL5 allele was associated with intra-familial phenotypic variability of the disease. However, both Weaving et al. [21] and Evans JC et al. [3] reported that twin sisters with the similar XCI pattern in cells from peripheral blood exhibited completely different clinical features. Hence, different phenotypes do not appear to be as a result of X-inactivation status. In this study, all the 9 females with *CDKL5* mutations have random XCI patterns and we may conclude that the differences of their clinical manifestations could not be attributed to XCI. Similar results are reported in the references [6,9]. It is worth noting that the XCI pattern in peripheral blood may not necessarily reflect the XCI pattern in neurons. Furthermore, the distribution of XCI in brain may differ from region to region due to its tissue-specific variation [32]. Therefore, in order to develop an effective relationship between the pattern of XCI and clinical manifestations, the above questions should be addressed first in future.

## Conclusions

This study is the first report of Chinese patients with *CDKL5* gene mutations. The common clinical features of the ten patients with *CDKL5* mutations include none or limited language development, severe hypotonia, deceleration of head growth, hand apraxia and hand stereotypies, as well as early-onset seizures presented initially as partial seizures and soon transformed to spasms. *CDKL5* mutation is an important cause in female patients with early onset epilepsy and severe psychomotor retardation without known etiology. In our study we cannot develop any clear relationship between the clinical manifestation and the type of *CDKL5* mutations as well as the XCI pattern. Complete screening of *CDKL5* exons including MLPA tests should be performed on both genders, although large rearrangement had not been detected in our cohort.

## Competing interests

The authors declare no conflict of interests.

## Authors’ contributions

XB obtained the funding and design the research. YZ, XZ, QZ, JZ, GC, JZ and JL acquired and provided clinical data and samples from patients. YZ and XZ carry out the laboratory work, QZ, JZ, GC, JZ and JL helped preparing the laboratory work. YZ analyzed most of the data and draft the manuscript. XB, LW, HP, XW revised the manuscript. All authors read and approved the final manuscript.

## Pre-publication history

The pre-publication history for this paper can be accessed here:

http://www.biomedcentral.com/1471-2350/15/24/prepub

## Supplementary Material

Additional file 1 **Online supplement detailed clinical manifestations of the patients with *CDKL5 *** **mutation in our cohort.** Click here for file
