# Supplementary material for: Clinical features and gene mutational spectrum of CDKL5-related diseases in a cohort of Chinese patients
Source: BMC Med Genet. 2014 Feb 25;15:24. doi: 10.1186/1471-2350-15-24 (PMC3938974; doi:10.1186/1471-2350-15-24)
Supplement: Additional file 1 — Online supplement detailed clinical manifestations of the patients with CDKL5 mutation in our cohort. [file 1471-2350-15-24-S1.doc]

Patient 1

The 2-year-old girl was diagnosed with early onset epileptic encephalopathy with a microdeletion c.1111 delC in exon12 of gene *CDKL5.* She was the first child of her non-consanguineous parents. The pregnancy was uneventful and she suffered from neonatal infection and metabolic acidosis after born at term. Her growth parameters were normal at delivery. At the age of 22 days, she started to have daily partial seizures, which remained resistant to multiple antiepileptic drugs. When she was 9 weeks old, the seizure type transformed to spasm. No improvement was achieved with the treatment of ACTH (adrenocorticotropic hormone) and ketogenic diet at 7-month. The seizure frequency was once every one or two days then. Her psychomotor development was significantly delayed. She can sit until 16 months, however, she couldn’t walk without help and speak. She had hand wringing and hand mouthing at 6 months. Her head circumference was 45 cm at one-year old. Physical examination showed severe hypotonia and EEG tests showed multifocal sharp and slow wave discharges without hypsarrhythmia even at the time of spasms presented. MRI was unremarkable. Urine, blood amino acid and organic acid were normal.

Patient 2

The patient with the Hanefeld variant of RTT is a 3-year-old girl who was born at term after an uneventful pregnancy from non-consanguineous healthy parents. She was indentified with splicing mutation ISV13+A>G of gene *CDKL5*. She had normal birth weight and head circumference. She was affected with partial seizures ten days after birth, and suffered from infantile spasms when she was 2-months Her psychomotor development was retarded. She could raise her head at 4 months and sit unaided at 11 months. . At two years old, she had three types of seizures including spasms, myoclonic seizures and tonic seizures. By that time, she could not walk, and could speak only one word. She could not sit and speak any words at age of 3. She had decreased muscle tone and strength. Her head circumference was 46.5 cm at two years old, and she presented hand stereotypies such as hand clapping, hand mouthing, without any purposeful hand using when she was 9 months old. At two months old, the EEG test showed hypsarrhythmia tendency. The follow-up EEGs showed atypical hypsarrhythmia, and the spasms, myoclonic seizures and tonic seizures were recorded during the Video-EEG test when she was two-year-old. Brain MRI was normal. Urine ,blood amino acid and organic acid level were normal. The seizures were poorly controlled by topiramate, valproate, ACTH and ketogenic diet.

Patient 3

The three-year old girl is the only child of her non-consanguineous parents, who are Han nationality with a frameshift mutation c.1791 ins G in exon12 of *CDKL5.* She was born at term with a history of possible hypoxia and normal growth parameters at birth. When she was 37 days old, she showed partial seizures. Atypical epileptic spasms appeared at 47 days, which remained resistant to multiple antiepileptic drugs. Hand stereotypies including hand wringing and hand mouthing appeared when she was 3 months old. Early onset epileptic encephalopathy was diagnosed. Her psychomotor development was severe delayed with hypotonia. She learned to roll at 6 month old, and to sit without support at 18 month old. But she did not develop any communication skills. At the age of two, she had profound mental retardation, poor eye contact and intermittent hand-wringing and hand-mouthing movements. Her head circumference was 46 cm at 2-year-old. EEG test at 3 months showed a few multifocal sharp-waves discharges without hypsarrhythmia though she had epileptic spasms [already](javascript:void(0)) at this moment. Brain MRI was normal. Urine and blood amino acid and organic acid were normal.

Patient 4

The patient with Hanefeld variant of RTT is a four-year-old girl with splicing mutation ISV6+1A>G who is the first child of her non-consanguineous parents from Han nationality. She was born at term with normal birth weight and head circumference. She showed partial seizures at one-hundred days and transformed to clusters of spasm 10 days later. Psychomotor development was severely delayed. She was able to sit till 24 months of age and never learned to walk without support. She could unconsciously say one word. She had hand stereotypic movements with hand wringing and hand mouthing. Heavy sigh and bruxism occurred at 1-year-old. Her head circumference was 46 cm at age of three-year-old. Muscle tone and strength are decreased mildly. EEG showed slow background and multifocal sharp, spike-waves discharges without hypsarrhythmia. An attack was recorded during the EEG manifested as partial seizure initially then evolved into spasms. Brain MRI was normal. Seizures were absent after 3 years old before which she took a combination of antiepileptic drugs including clonazepam, topiramate, valproate and lamotrigine.

Patient 5

The five-year-old girl with the Hanefeld variant of RTT is the only child of her non-consanguineous parents. She was diagnosed with c.1375C>T mutation in exon12 of *CDKL5* gene. She had normal delivery history. Her growth parameters also were normal at birth. She showed partial seizures at age of 30 days and suffered from spasms when she was one and a half years. She could sit unaided at 6-month-old. After then, she had psychomotor delay. She could walk a few steps until 4 years with hypotonia. She did not develop any communication skill, and she lost the capability with her hands and showed intermittent hand wringing movements. Her head circumference was 46.5 cm at 3-year-old. Her EEG showed multifocal spikes, sharp-waves, spike-waves discharges as [hypsarrhythmia](javascript:void(0)) when she was 19 months. Brain MRI was normal. The seizures were poorly controlled by numerous antiepileptic drugs.

Patient 6 and Patient 7

The twin sisters were 2 years and 6months old when the elder sister was referred to our hospital. Their parents are non-consanguineous and healthy adults. They were identified with the same frameshift mutation of c.891_892ins TT in exon11 of *CDKL5* gene. The twins were cesarean delivered at 39 gestation weeks. The elder sister started to have partial seizures at 10 weeks of her life, with eyes blinking first and then rolling, limbs flexion, 2 or 3 times a day, then transformed to epileptic spasm resistant to multiple antiepileptic drugs at 3 months old. Her developmental milestones were profoundly delayed. She could raise her head reluctantly at 3 months and sit with aid at 6 month. At present, she couldn’t walk at all and didn’t acquire any language skills except non-purposeful murmurs of “pa ma”. She developed intermittent hand wringing, without any purposeful hand use. Her head circumference was 46.5 cm at 1-year then followed by decreased head growth. Hyperventilation and breath holding presented at 6 months. Her EEG showed [hypsarrhythmia](javascript:void(0)) at two and half years old. A cluster of spasms was recorded during the EEG test. Brain CT and urine metabolic screening test at 3-month old were unremarkable. When the elder sister was ascertained to carry the *CDKL5* mutation (c.891_892 ins TT), we performed the gene test on the younger twin sister and detected the same mutation. The younger twin sister had the similar phenotype including the age of seizure onset, the types, severity and frequency of the seizures, the profound cognitive impairment and some Rett-like features such as hand stereotypies. The EEG and brain image of the younger sister were not available.

Patient 8

The five-month-old boy is the first child of a non-consanguineous parents with a missense mutation of c.533G>A in exon8 of *CDKL5* gene. His mother had taken progesterone to prevent miscarriages during the first three months of pregnancy. He started to have partial seizure in the 2nd month after his normal delivery. Several days later, he was attacked by clusters of epileptic spasms and was diagnosed with infantile spasms. The frequent seizures hampered the boy’s psychomotor development severely. Actually he could not raise his head or babble when he was 5 months. He presented autistic features such as poor eye contact and poor response to amusement after seizure onset and without obvious hand stereotypies such as hand wringing, hand washing and hand mouthing. Physical and neurological examination revealed microcephaly with head circumference of 40cm and apparent hypotonia. The EEG showed atypical hypsarrhythmia. Clusters of spasms were recorded during the Video-EEG test. Brain MRI at 2-month-old was unremarkable. Urine metabolic screening was normal. The seizures were resistant to multiple antiepileptic drugs. He still has daily cluster spasms now.

Patient 9

The 13-month-old girl is the first child of a non-consanguineous Han Chinese couple with micro-deletion of c.2360delA in exon16 of *CDKL5* gene. Thirty five days after an uneventful pregnancy and birth, she suffered from partial seizures, with her head turning to right, eyes gazing, face flushing and asymmetric tonic in her arms and legs. Seizures lasted 20 seconds to 5 minute, occurring 5-6 times daily. Her head circumference was 45cm. She had severe psychomotor developmental delay with hypotonia. She could not raise her head steadily and sit without aid. Meanwhile, she had much more difficulty in swallowing. She showed autistic characters and hand stereotypies with hand wringing and hand mouthing in most of daily life, without any useful hand skills. Heavy sigh and bruxism occurred at 1-year-old. She did not develop any communication skills and could not speak a single word. She was suspected to be the Hanefeld variant of RTT. EEG at 10-month-old showed bilateral frontal and anterior temporal spike and slow waves. Brain MRI was normal at 1-year-old. Phenobarbital was administrated as initial treatment and discontinued for limited effect, then valproate and [levetiracetam](app:ds:levetiracetam) started. Alhough several antiepileptic drugs were administrated, the seizures persisted without any improvements.

Patient 10

The girl was referred to us at the age of 2-year and 9-month and was indentified with micro-deletion of c.234delA in exon5 of *CDKL5* gene. After an uneventful pregnancy, she was born with a history of hypoxia. At the age of 40 days, she suffered from partial seizures, which presented as inflexion of her arms and legs, face flushing and eyes gazing lasting 4-5 seconds. The seizures occurred 7-8 times per day. [Topiramate](app:ds:topiramate), valproate and [levetiracetam](app:ds:levetiracetam) were administrated. The seizures become intermittent due to the irregular administration of drugs. At the age of 2 years and a half, the seizures transformed to spasm with head nodding, arm flexion occurred in clusters. At 2 years and 10 months old, myoclonic seizures, isolated and clusters of spasms, atypical absence seizures were recorded during V-EEG test. Early-onset seizure variant of RTT was diagnosed .The girl had profound psychomotor delay. She did not acquire the ability of eye tracking until 22-month, and could raise her head steadily at 2-year and 4-months. She could not sit without aid and walk. Purposeful hand skills and communicative language were never acquired. Scoliosis was found at 6 months. Hand washing and hand wringing occurred at 7 months. Bruxism presented when she was 1-year-old. The V-EEG at 8 months showed spikes, spike-waves discharged at bilateral Rolandic area. At 2-year and 11-month, repeated EEG showed multifocal and generalized high amplitude slow waves, sharp-waves, spikes, spike-waves, multi-spike-waves. Brain MRI was normal.
